# Impact of Air Pollution on Chronic Airway Narrowing

**DOI:** 10.7759/cureus.88135

**Published:** 2025-07-17

**Authors:** Pavlos Vongelis, Petros Bakakos, Nikoleta Rovina, Nikolaos Koulouris

**Affiliations:** 1 First Department of Respiratory Medicine, Medical School, Sotiria Hospital, National and Kapodistrian University of Athens, Athens, GRC

**Keywords:** asthma, bronchiectasis, carbon monoxide (co), chronic airway narrowing, copd, nitrogen dioxide (no2), ozone (o3), particulate matter (pm10), respiratory diseases, sulfur dioxide (so2)

## Abstract

Introduction: The increasing availability of data from electronic health records in healthcare organizations and systems has made it feasible to assess the relationship between various environmental parameters (e.g., pollution levels, meteorological data), hospital admissions, morbidity, and mortality associated with lung diseases. This study aimed to assess the correlation between air pollution levels and respiratory function parameters in patients with chronic obstructive lung diseases residing in various regions of the Attica Basin, Greece.

Materials and methods: Air pollution measurements were obtained from the Department of Atmospheric Quality of the Greek Ministry of Environment and Energy. In particular, data from daily bulletins and air pollution measurements were utilized. Numerical averages were calculated across all monitoring stations.

Results: The analysis revealed statistically significant negative correlations between the concentrations of tropospheric ozone (O₃), sulfur dioxide (SO₂), nitrogen dioxide (NO₂), carbon monoxide (CO), and particulate matter (PM₁₀) and the respiratory parameters forced vital capacity (FVC%pred), forced expiratory volume in one second (FEV₁%pred), forced mid-expiratory flow between 25 and 75% of FVC (FEF_25-75%_pred), and peak expiratory flow (PEF%pred) on the day of examination (Day 0).

Conclusions: Air pollution measurements were found to be significantly and negatively correlated with lung function parameters. Specifically, increased concentrations of O_3_, CO, SO_2_, NO_2_, and PM_10_ were significantly associated with lower values of the measured lung function parameters. Climate change may significantly affect future levels of O_3_ and the other air pollutants studied in this work. Many regions may face substantial increases in their concentrations, despite the implementation of emission reduction measures. This study clearly highlights the adverse effects of the examined air pollutants on key lung function parameters associated with obstructive lung diseases.

## Introduction

Ambient air pollution is widely recognized as a significant threat to human health [1]. Numerous epidemiological studies conducted globally have demonstrated strong associations between short-term increases in air pollution levels and a wide range of respiratory and cardiovascular conditions, including asthma [2], exacerbations of chronic obstructive pulmonary disease (COPD) [3], stroke, and ischemic heart disease [4,5].

Although several mechanisms have been proposed to explain these adverse health effects [6], there is still limited consensus on which specific components of air pollution are the most harmful [7]. Environmental exposures, such as ambient air pollution, contaminated water and food, soil pollutants, and occupational contact with toxic substances, are well-established contributors to the global disease burden [8-10]. These exposures are known to induce oxidative stress, inflammation, mitochondrial dysfunction, endocrine disruption, and a range of genetic and epigenetic modifications, all of which may contribute to the pathogenesis of chronic diseases [11-13].

Inhalation of particulate matter (PM), a complex mixture of solid and liquid particles suspended in the air, can lead to the generation of reactive oxygen species in lung epithelial cells, triggering inflammatory responses, apoptosis, and tissue injury [14-16]. Moreover, air pollutants can induce DNA methylation, somatic mutations, and histone modifications, mechanisms increasingly implicated in the development of malignancies and chronic pulmonary disorders [17,18].

The respiratory system is particularly susceptible to these effects, as pollutant-induced immune dysregulation, viral reactivation, and alterations in the lung microbiome contribute to increased vulnerability to infections such as bronchitis and pneumonia, as well as to the exacerbation of preexisting respiratory conditions [8]. Importantly, the systemic effects of air pollution have been well documented, including its involvement in the development and progression of cardiovascular, cerebrovascular, and metabolic diseases, as well as various types of cancer [8,19].

According to the World Health Organization (WHO), an estimated 12.6 million deaths annually are attributable to environmental risk factors [20], with air pollution identified as a major contributor. PM alone has been linked to approximately 9 million premature deaths worldwide [21].

Key air pollutants associated with these adverse health outcomes include ozone (O_3_), carbon monoxide (CO), nitrogen oxides (NOx and NO_2_), sulfur dioxide (SO_2_), and PM [22]. These substances are routinely monitored to assess air quality and evaluate associated public health risks. Indoor air pollution, primarily resulting from the use of solid fuels and biomass for heating and cooking, also remains a significant contributor to global morbidity and mortality [22].

The aim of this analysis is to investigate the correlation between air pollution levels and respiratory function parameters in patients with chronic obstructive lung diseases residing in various regions of the Attica Basin, Greece.

## Materials and methods

**Figure 1:**
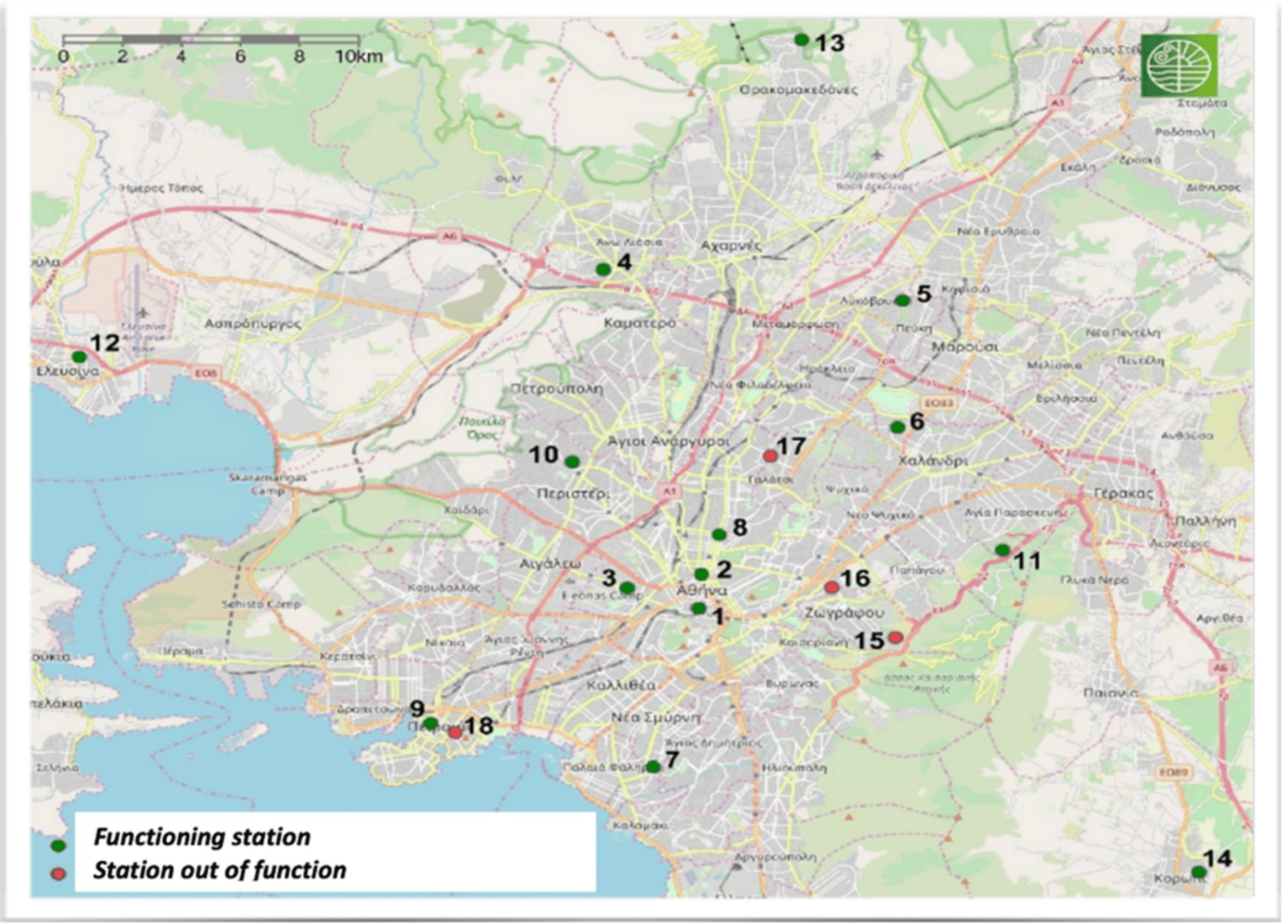
Map of the atmospheric pollution monitoring stations of the Greek Ministry of Environment and Energy in the Attica Basin. Source: Ministry of Environment and Energy, Athens [23].

Environmental pollution data were obtained from the Department of Atmospheric Quality of the Greek Ministry of Environment and Energy, specifically from daily bulletins and other air pollution measurement records. Subsequently, the numerical average was calculated from all the monitoring stations, which include all the completeness criteria for each calendar day of the year, for the following pollutants: O₃, nitrogen dioxide (NO₂), SO₂, CO, and suspended particulate matter (PM₁₀). Measurements from the same monitoring stations were consistently used throughout the study period, from October 2021 to June 2023. The locations of these monitoring stations are presented in Figure 1.

The following criteria were applied to ensure data completeness for each measuring station:

Given that the highest levels of ozone (O₃) typically occur during the day and that ozone is a photochemical pollutant, it was necessary to have at least 75% of hourly values recorded from early morning until 7:00 p.m. in order to determine the daily maximum O₃ concentrations. For the remaining pollutants, daily concentrations were only considered valid if at least 75% of hourly values were available for each day of the year. Any station with more than 25% missing data during the study period was excluded from the analysis. In cases where a station had a deficiency for a specific pollutant, the average from the remaining stations in Attica was statistically correlated with the rest, excluding the deficient station from the specific analysis.

In the next step, a detailed review of data distributions, descriptive statistics, and measurement tables was conducted. To assess potential associations between air pollutant concentrations and respiratory function, data were analyzed using appropriate autocorrelation methods, specifically employing Pearson’s parametric correlation tests.

To assess and monitor respiratory conditions, patients diagnosed with bronchial asthma, COPD, and bronchiectasis were evaluated at the Respiratory Function Laboratory of the First Department of Respiratory Medicine, School of Medicine, National and Kapodistrian University of Athens. These were consecutive patients on their follow-up visits to the lab for evaluating their treatment. A power calculation was conducted, and 145 patients were needed for the statistical power of the study. There was control for potential confounding factors, and patients with confounding factors were excluded (occupational factors, inhabitant factors, medications). 

After recording the patients’ date of arrival, gender, age, weight, height, and smoking status, the relevant data were entered into a database using appropriate interdependence models. Subsequently, volume-time and flow-volume curves were generated via standard spirometry to assess pulmonary function. The parameters measured included: forced expiratory volume in one second (FEV₁, in liters [L] and % predicted), forced vital capacity (FVC, [L] and % predicted), FEV₁/FVC ratio (%), forced mid-expiratory flow between 25 and 75% of FVC (FEF_25-75%_, [L/s] and % predicted), and peak expiratory flow (PEF, [L/s] and % predicted). 

Lung function tests were performed during regular follow-up of stable patients. The day of spirometry was defined as Day 0, which was the same day the measurements of pollution were derived. Approval for the study was obtained from the Bioethics and Ethics Committee of the School of Medicine of the University of Athens (Application number: 382/5-11-2020). 

Statistical processing of data

Parametric Pearson correlation tests were conducted using IBM SPSS Statistics for Windows, Version 30.0 (Released 2024; IBM Corp., Armonk, New York, USA) to assess the correlation between the parameters forced vital capacity (FVC%pred), forced expiratory volume in one second (FEV₁%pred), forced mid-expiratory flow (FEF_25-75%_pred), peak expiratory flow (PEF%pred), and measurements of atmospheric pollutants in the Attica Basin on the day of each patient's examination. The analysis of lung function test values (as also in healthy adults) showed a normal distribution, allowing for the use of parametric correlation tests.

The total sample consisted of 210 patients diagnosed with various chronic obstructive lung diseases. The study period for patients' evaluation was from October 2021 to June 2023. All patients were stable regarding disease state and proceeded for spirometry due to regular follow-up from the attending physician.

The findings are structured into three main sections: (a) The first section examines the aforementioned correlations in the overall daily sample. (b) The second section analyzes these correlations separately for each gender on a daily basis. (c) The third section investigates the correlations based on smoking status (smokers vs. non-smokers), also on a daily basis.

The mean ± standard deviation (SD), along with the range of values, was used to describe patient characteristics and study parameters. In the correlation tables, respiratory function parameters are listed in rows, while atmospheric pollutants are listed in columns.

## Results

Overall sample correlation

A total of 210 consecutive patients with various chronic obstructive lung diseases were studied. Demographic and clinical characteristics of the study population, stratified by sex and smoking status, are presented in Table 1.

**Table 1:** Demographic and clinical characteristics of patients (mean value ± SD). FVC: forced vital capacity; FEV₁: forced expiratory volume in one second; FEF_25-75%_: forced mid-expiratory flow between 25 and 75% of FVC; PEF: peak expiratory flow.

	Overall	Males	Females	Smokers	Non-smokers
Subgroup (n, %)	210 (100%)	119 (56.7%)	91 (43.3%)	125 (59.5%)	85 (40.5%)
Age	57.82 ± 14.43	60.23 ± 12.11	54.67 ± 11.98	61.89 ± 15.01	51.83 ± 13.31
FVC (L)	3.491 ± 1.269	3.931 ± 1.361	2.916 ± 0.849	3.581 ± 1.332	3.359 ± 1.166
FVC (%pred)	74.128% ± 9.774	71.084% ± 11.236	78.109% ± 6.982	73.792% ± 11.244	74.622% ± 7.369
FEV₁ (L)	2.453 ± 0.968	2.728 ± 1.077	2.094 ± 0.652	2.482 ± 1.066	2.411 ± 0.808
FEV₁ (%pred)	68.019% ± 11.610	65.529% ± 13.692	71.274% ± 8.154	65.400% ± 13.887	71.870% ± 6.818
FEV₁/FVC (%)	68.361% ± 11.667	68.443% ± 13.382	68.253% ± 8.684	68.126% ± 13.071	68.706% ± 8.705
FEF_25-75%_ (L/s)	2.210 ± 1.297	2.438 ± 1.463	1.911 ± 0.972	2.088 ± 1.363	2.389 ± 1.179
FEF_25-75%_ (%pred)	50.380% ± 20.278	52.495% ± 23.458	47.615% ± 15.447	45.328% ± 19.331	57.809% ± 20.284
PEF (L/s)	6.824 ± 2.481	7.650 ± 2.778	5.743 ± 1.447	6.908 ± 2.827	6.700 ± 1.868
PEF (%pred)	72.185% ± 15.115	71.462% ± 18.854	73.131% ± 9.276	69.768% ± 18.665	75.739% ± 8.259

The average age of the participants was 57.82 ± 14.43 years, with 56.7% of them being men (n = 119) and 43.3% of them being women (n = 91). Regarding respiratory function parameters, the mean FVC for the overall sample was 3.491 ± 1.269 L, with a mean FVC%pred of 74.128% ± 9.774. The mean FEV₁ was 2.453 ± 0.968 L, with a mean FEV_1_%pred of 68.019% ± 11.610. The FEV₁/FVC% ratio was 68.361% ± 11.667, indicating an obstructive syndrome. Additionally, the mean FEF_25-75% _was 2.210 ± 1.297 L/second, with a mean FEF_25-75%_ (%pred) of 50.380% ± 20.278. The mean PEF was 6.824 ± 2.481 L/second, and the average PEF%pred was 72.185% ± 15.115.

Correlation Between Pulmonary Function Indices and Air Pollution Levels in the Overall Sample on the Day of Examination (Day 0)

According to Table 2, the variable O₃ correlates negatively and statistically significantly with the FVC%pred parameter. In other words, increased values of O₃ are significantly associated with lower values of forced vital capacity (FVC%pred), (r = -0.781, p = 0.032). Additionally, O₃ is negatively and statistically significantly correlated with the FEF_25-75%_ (%pred) parameter, indicating that higher values of the pollutant O₃ are significantly related to lower values of the forced expiratory flow between 25 and 75% of the FVC test (r = -0.789, p = 0.012). O₃ is also negatively correlated with the PEF%pred (r = -0.761, p = 0.010) and the FEV₁%pred (r = -0.788, p = 0.034) in a statistically significant manner.

**Table 2:** Correlations on the day of examination (Day 0) between FVC%pred, FEV₁%pred, FEF25-75%pred, and PEF%pred and air pollutants (O₃, NO₂, SO₂, CO, PM₁₀) in all patients. FVC (%pred): forced vital capacity as percentage of predicted; FEV₁ (%pred): forced expiratory volume in one second as percentage of predicted; FEF_25-75%_ (%pred): forced mid-expiratory flow between 25 and 75% of FVC as percentage of predicted; PEF (%pred): peak expiratory flow as percentage of predicted; O_3_ (Day 0) [μg/m³]: ozone concentration on the day of examination; NO₂ (Day 0) [μg/m³]: nitrogen dioxide concentration on the day of examination; SO₂ (Day 0) [μg/m³]: sulfur dioxide concentration on the day of examination; CO (Day 0) [mg/m³]: carbon monoxide concentration on the day of examination; PM₁₀ (Day 0) [μg/m³]: particulate matter with aerodynamic diameter ≤10 μm on the day of examination; n = 210 patients; p-value (**p < 0.05).

Air pollutants		FVC (%pred)	FEV_1_ (%pred)	FEF_25-75% _(%pred)	PEF (%pred)
O₃ (Day 0) [μg/m³]	r	-0.781	-0.788	-0.789	-0.761
	p	0.032^**^	0.034^**^	0.012^**^	0.010^**^
NO₂ (Day 0) [μg/m³]	r	-0.682	-0.704	-0.670	-0.519
	p	0.029^**^	0.030^**^	0.047^**^	0.072
SO₂ (Day 0) [μg/m³]	r	-0.761	-0.671	-0.507	-0.560
	p	0.032^**^	0.019^**^	0.069	0.083
CO (Day 0) [mg/m³]	r	-0.508	-0.774	-0.699	-0.365
	p	0.055	0.025^**^	0.020^**^	0.107
PM₁₀ (Day 0) [μg/m³]	r	-0.701	-0.320	-0.481	-0.682
	p	0.017^**^	0.064	0.081	0.033^**^

In reference to NO₂, statistically significant negative correlations were found with the FVC%pred (r = -0.682, p = 0.029), the FEV₁%pred (r = -0.704, p = 0.030), and the FEF_25-75%_ (%pred) parameter (r = -0.670, p = 0.047). For SO₂, statistically significant negative correlations were found with the FVC%pred (r = -0.761, p = 0.032) and the FEV₁%pred (r = -0.671, p = 0.019). Regarding CO, a statistically significant negative correlation was observed with the FEV₁%pred (r = -0.774, p = 0.025) and also with the FEF_25-75%_ (%pred) parameter (r = -0.699, p = 0.020). In the case of PM₁₀ atmospheric particles, statistically significant negative correlations were observed with the FVC%pred (r = -0.701, p = 0.017) and the PEF%pred (r = -0.682, p= 0.033).

Correlation among male participants

A total of 210 patients were included in the study, 56.7% of whom were men (n = 119). Among the male participants, the mean age was 60.23 ± 12.11 years. The mean FVC was 3.931 ± 1.361 L, with a mean FVC%pred of 71.084% ± 11.236. The mean FEV₁ was 2.728 ± 1.077 L, with a mean FEV₁%pred of 65.529% ± 13.692. The FEV₁/FVC% ratio was 68.443% ± 13.382, indicating the presence of obstructive lung syndrome. Additionally, the mean FEF_25-75%_ was 2.438 ± 1.463 L/second, with a corresponding FEF_25-75%_​ ​​​​​​(%pred) of 52.495% ± 23.458. The mean PEF was 7.650 ± 2.778 L/second, while the mean PEF%pred was 71.462% ± 18.854.

Correlation Between Pulmonary Function Indices and Air Pollution Levels in Males on the Day of Examination (Day 0)

According to Table 3, the variable O₃ was negatively and statistically significantly correlated with FEF_25-75%_ (%pred). In other words, increased O₃ values are significantly associated with lower values of forced mid-expiratory flow between 25 and 75% of the FVC test (r = -0.787, p = 0.040). O₃ also demonstrated a statistically significant negative correlation with the FVC%pred parameter (r = -0.769, p = 0.009). Furthermore, statistically significant negative correlations were observed between O_3_ and FEV₁%pred (r = -0.741, p = 0.011), as well as between O₃ concentrations and PEF%pred values (r= -0.725, p= 0.019).

**Table 3:** Correlations on the day of examination (Day 0) between FVC%pred, FEV₁%pred, FEF25-75​​​​​​%pred, and PEF%pred and air pollutants (O₃, NO₂, SO₂, CO, PM₁₀) in men. FVC (%pred): forced vital capacity as percentage of predicted; FEV₁ (%pred): forced expiratory volume in one second as percentage of predicted; FEF_25-75%_ (%pred): forced mid-expiratory flow between 25 and 75% of FVC as percentage of predicted; PEF (%pred): peak expiratory flow as percentage of predicted; O₃ (Day 0) [μg/m³]: ozone concentration on the day of examination; NO₂ (Day 0) [μg/m³]: nitrogen dioxide concentration on the day of examination; SO₂ (Day 0) [μg/m³]: sulfur dioxide concentration on the day of examination; CO (Day 0) [mg/m³]: carbon monoxide concentration on the day of examination; PM₁₀ (Day 0) [μg/m³]: particulate matter with aerodynamic diameter ≤10 μm on the day of examination; n = 119 patients; p-value (**p < 0.05, ***p < 0.01).

Air pollutants		FVC (%pred)	FEV_1 _(%pred)	FEF_25-75% _(%pred)	PEF (%pred)
O₃ (Day 0) [μg/m³]	r	-0.769	-0.741	-0.787	-0.725
	p	0.009^***^	0.011^**^	0.040^**^	0.019^**^
NO₂ (Day 0) [μg/m³]	r	-0.691	-0.720	-0.698	-0.399
	p	0.023^**^	0.015^**^	0.028^**^	0.106
SO₂ (Day 0) [μg/m³]	r	-0.721	-0.675	-0.390	-0.575
	p	0.011^**^	0.029^**^	0.109	0.287
CO (Day 0) [mg/m³]	r	-0.678	-0.791	-0.657	-0.344
	p	0.025^**^	0.008^***^	0.013^**^	0.190
PM₁₀ (Day 0) [μg/m³]	r	-0.724	-0.389	-0.089	-0.678
	p	0.048^**^	0.093	0.130	0.014^**^

In the case of NO₂, statistically significant negative correlations were found primarily with FEV₁%pred (r = -0.720, p = 0.015), FVC%pred (r = -0.691, p = 0.023), and FEF_25-75​​​​​​%_ (%pred) (r = -0.698, p = 0.028). SO₂ was also found to have statistically significant negative correlations with FEV₁%pred (r = -0.675, p = 0.029) and FVC%pred (r = -0.721, p = 0.011). These findings suggest that higher SO₂ levels are significantly associated with reduced FVC%pred values. For CO, statistically significant negative correlations were observed with FEV₁%pred (r = -0.791, p = 0.008), FEF_25-75%_ (%pred) (r = -0.657, p = 0.013), and FVC%pred (r = -0.678, p = 0.025). In other words, increased CO concentrations are significantly associated with reduced values in FVC%pred, FEV₁%pred, and FEF_25-75​​​​​​%_ (%pred). Regarding PM₁₀ air particles, statistically significant negative correlations were found with both the FVC%pred parameter (r = -0.724, p = 0.048) and the PEF%pred parameter (r = -0.678, p = 0.014).

Correlation among female participants

Among the female patients, who make up 43.3% (n=91) of the sample, the mean age was 54.67 ± 11.98 years. The mean FVC was 2.916 ± 0.849 L, with a mean FVC%pred of 78.109% ± 6.982. The mean FEV₁ was 2.094 ± 0.652 L, with a mean FEV₁%pred of 71.274% ± 8.154. The FEV₁/FVC% ratio was 68.253% ± 8.684, indicating the presence of obstructive lung syndrome. Additionally, the mean FEF_25-75​​​​​​%_ was 1.911 ± 0.972 L/second, with a corresponding FEF_25-75%_​​​ ​​​​​​​​​​​(%pred) of 47.615% ± 15.447. The mean PEF was 5.743 ± 1.447 L/second, while the mean PEF%pred was 73.131% ± 9.276.

Correlation Between Pulmonary Function Indices and Air Pollution Levels in Females on the Day of Examination (Day 0)

In Table 4, we noted that the O₃ variable is negatively and statistically significantly correlated with the FEF_25-75%​​​​​​​​​​​​​​ _(%pred) parameter, i.e., increased values of O₃ are significantly related to low values of the forced expiratory flow between 25 and 75% of the FVC%pred test (r = -0.774, p = 0.011). In addition, O₃ correlates negatively and statistically significantly with the FVC%pred parameter (r = -0.767, p = 0.029) and the FEV₁%pred (r = -0.775, p = 0.036). Furthermore, O₃ shows a statistically significant negative correlation with the PEF%pred (r = -0.740, p = 0.015).

**Table 4:** Correlations on the day of examination (Day 0) between FVC%pred, FEV₁%pred, FEF25-75%​​​​​​​pred, and PEF%pred and air pollutants (O₃, NO₂, SO₂, CO, PM₁₀) in women. FVC (%pred): forced vital capacity as percentage of predicted; FEV₁ (%pred): forced expiratory volume in one second as percentage of predicted; FEF_25-75%_ (%pred): forced mid-expiratory flow between 25 and 75% of FVC as percentage of predicted; PEF (%pred): peak expiratory flow as percentage of predicted; O₃ (Day 0) [μg/m³]: ozone concentration on the day of examination; NO₂ (Day 0) [μg/m³]: nitrogen dioxide concentration on the day of examination; SO₂ (Day 0) [μg/m³]: sulfur dioxide concentration on the day of examination; CO (Day 0) [mg/m³]: carbon monoxide concentration on the day of examination; PM₁₀ (Day 0) [μg/m³]: particulate matter with aerodynamic diameter ≤10 μm on the day of examination; n = 91 patients; p-value (**p < 0.05).

Air pollutants		FVC (%pred)	FEV_1_ (%pred)	FEF_25-75% _(%pred)	PEF (%pred)
O₃ (Day 0) [μg/m³]	r	-0.767	-0.775	-0.774	-0.740
	p	0.029^**^	0.036^**^	0.011^**^	0.015^**^
NO₂ (Day 0) [μg/m³]	r	-0.703	-0.476	-0.687	-0.301
	p	0.040^**^	0.066	0.018^**^	0.502
SO₂ (Day 0) [μg/m³]	r	-0.702	-0.687	-0.427	-0.314
	p	0.012^**^	0.032^**^	0.082	0.161
CO (Day 0) [mg/m³]	r	-0.409	-0.716	-0.671	-0.064
	p	0.061	0.029^**^	0.028^**^	0.320
PM₁₀ (Day 0) [μg/m³]	r	-0.712	-0.511	-0.338	-0.679
	p	0.036^**^	0.121	0.118	0.021^**^

Two statistically significant negative correlations were found for NO₂: one with the FEF_25-75%_​​​​​​​ (%pred) (r = -0.687, p = 0.018) and another with the FVC%pred (r = -0.703, p = 0.040). SO₂ was found to have statistically significant negative correlations with the FEV₁%pred (r = -0.687, p = 0.032) and FVC%pred (r = -0.702, p = 0.012) parameters. Regarding CO, statistically significant negative correlations were found with the FEV₁%pred (r = -0.716, p = 0.029) and the FEF_25-75​​​​​​%_ (%pred) (r = -0.671, p = 0.028). After processing the data on PM₁₀ air particles, statistically significant negative correlations were found between the PEF%pred (r = -0.679, p= 0.021) and the FVC%pred parameter (r = -0.712, p = 0.036).

Correlation among smokers

Among the smokers, who constitute 59.5% (n=125) of the sample, the mean age was 61.89 ± 15.01 years. The mean FVC was 3.581 ± 1.332 L, with a mean FVC%pred of 73.792% ± 11.244. The mean FEV₁ was 2.482 ± 1.066 L, with a mean FEV₁%pred of 65.400% ± 13.887. The FEV₁/FVC% ratio was 68.126% ± 13.071, indicating the presence of obstructive lung syndrome. Additionally, the mean FEF_25-75​​​​​​%_ was 2.088 ± 1.363 L/second, with a corresponding FEF_25-75%​​​​​​​ _(%pred) of 45.328% ± 19.331. The mean PEF was 6.908 ± 2.827 L/second, while the mean PEF%pred was 69.768% ± 18.665.

Correlation Between Pulmonary Function Indices and Air Pollution Levels in Smokers on the Day of Examination (Day 0)

Considering Table 5, we observe that the O₃ variable is negatively and statistically significantly correlated with the FEF_25-75%_​​​​​​​ (%pred), i.e., increased values of O₃ are significantly accompanied by low values of the forced expiratory flow between 25 and 75% of the FVC%pred test (r = -0.786, p = 0.016). In addition, O₃ correlates negatively and statistically significantly with the FVC%pred parameter (r = -0.779, p = 0.021). Additionally, statistically significant negative correlations were found between O₃ and FEV₁%pred test values (r = -0.774, p = 0.031) and between O₃ concentrations and PEF%pred test values (r = -0.748, p = 0.029).

**Table 5:** Correlations on the day of examination (Day 0) between FVC%pred, FEV₁%pred, FEF25-75%pred, and PEF%pred and air pollutants (O₃, NO₂, SO₂, CO, PM₁₀) in smokers. FVC (%pred): forced vital capacity as percentage of predicted; FEV₁ (%pred): forced expiratory volume in one second as percentage of predicted; FEF_25-75%_ (%pred): forced mid-expiratory flow between 25 and 75% of FVC as percentage of predicted; PEF (%pred): peak expiratory flow as percentage of predicted; O₃ (Day 0) [μg/m³]: ozone concentration on the day of examination; NO₂ (Day 0) [μg/m³]: nitrogen dioxide concentration on the day of examination; SO₂ (Day 0) [μg/m³]: sulfur dioxide concentration on the day of examination; CO (Day 0) [mg/m³]: carbon monoxide concentration on the day of examination; PM₁₀ (Day 0) [μg/m³]: particulate matter with aerodynamic diameter ≤10 μm on the day of examination; n = 125 patients; p-value (**p < 0.05).

Air pollutants		FVC (%pred)	FEV_1_ (%pred)	FEF_25-75% _(%pred)	PEF (%pred)
O₃ (Day 0) [μg/m³]	r	-0.779	-0.774	-0.786	-0.748
	p	0.021^**^	0.031^**^	0.016^**^	0.029^**^
NO₂ (Day 0) [μg/m³]	r	-0.696	-0.741	-0.684	-0.648
	p	0.027^**^	0.015^**^	0.025^**^	0.097
SO₂ (Day 0) [μg/m³]	r	-0.751	-0.679	-0.354	-0.731
	p	0.034^**^	0.017^**^	0.055	0.172
CO (Day 0) [mg/m³]	r	-0.717	-0.723	-0.661	-0.257
	p	0.134	0.012^**^	0.039^**^	0.246
PM_10_ (Day 0) [μg/m³]	r	-0.705	-0.614	-0.813	-0.670
	p	0.017^**^	0.761	0.088	0.020^**^

For NO₂, statistically significant negative correlations were found with the parameters FVC%pred (r = -0.696, p = 0.027), FEV₁%pred (r = -0.741, p = 0.015), and FEF_25-75%​​​​​​​_ (%pred) (r = -0.684, p = 0.025). SO₂ showed statistically significant negative correlations with the parameters FVC%pred (r = -0.751, p = 0.034) and FEV₁%pred (r = -0.679, p = 0.017). Regarding CO, statistically significant negative correlations were found with the FEV₁%pred (r= -0.723, p= 0.012) and FEF_25-75​​​​​​%_ (%pred) (r = -0.661, p = 0.039). After processing the data, statistically significant negative correlations were found with the parameters PEF%pred (r = -0.670, p = 0.020) and FVC%pred (r = -0.705, p = 0.017) for air particles PM₁₀.

Correlation among non-smokers

Among the non-smokers, who constitute 40.5% (n = 85) of the sample, the mean age was 51.83 ± 13.31 years. The mean FVC was 3.359 ± 1.166 L, with a mean FVC%pred of 74.622% ± 7.369. The mean FEV₁ was 2.411 ± 0.808 L, with a mean FEV₁%pred of 71.870% ± 6.818. The FEV₁/FVC% ratio was 68.706% ± 8.705, indicating the presence of obstructive lung syndrome. Additionally, the mean FEF_25-75%_​​​​​​​ was 2.389 ± 1.179 L/second, with a corresponding FEF_25-75​​​​​​%​​​​​​​_ (%pred) of 57.809% ± 20.284. The mean PEF was 6.700 ± 1.868 L/second, while the mean PEF%pred was 75.739% ± 8.259.

Correlation Between Pulmonary Function Indices and Air Pollution Levels in Non-smokers on the Day of Examination (Day 0)

Considering Table 6, we found a statistically significant negative correlation (r = -0.786, p = 0.003) between the variable O_3_ and the parameter FEF_25-75% _(%pred). Simultaneously, statistically significant negative correlations were found between O_3_ and the FVC%pred (r = -0.797, p = 0.009). Additionally, statistically significant negative correlations were observed between the O_3_ and the FEV_1_%pred (r = -0.789, p = 0.007) as well as between O₃ concentrations and PEF%pred (r = -0.792, p = 0.011).

**Table 6:** Correlations on the day of examination (Day 0) between FVC%pred, FEV₁%pred, FEF25-75%pred, and PEF%pred and air pollutants (O₃, NO₂, SO₂, CO, PM₁₀) in non-smokers. FVC (%pred): forced vital capacity as percentage of predicted; FEV₁ (%pred): forced expiratory volume in one second as percentage of predicted; FEF_25-75%_ (%pred): forced mid-expiratory flow between 25 and 75% of FVC as percentage of predicted; PEF (%pred): peak expiratory flow as percentage of predicted; O₃ (Day 0) [μg/m³]: ozone concentration on the day of examination; NO₂ (Day 0) [μg/m³]: nitrogen dioxide concentration on the day of examination; SO₂ (Day 0) [μg/m³]: sulfur dioxide concentration on the day of examination; CO (Day 0) [mg/m³]: carbon monoxide concentration on the day of examination; PM₁₀ (Day 0) [μg/m³]: particulate matter with aerodynamic diameter ≤10 μm on the day of examination; n = 85 patients; p-value (**p < 0.05, ***p < 0.01).

Air pollutants		FVC (%pred)	FEV_1_ (%pred)	FEF_25-75% _(%pred)	PEF (%pred)
O₃ (Day 0) [μg/m³]	r	-0.797	-0.789	-0.786	-0.792
	p	0.009^***^	0.007^***^	0.003^***^	0.011^**^
NO₂ (Day 0) [μg/m³]	r	-0.745	-0.773	-0.571	-0.551
	p	0.019^**^	0.017^**^	0.211	0.065
SO₂ (Day 0) [μg/m³]	r	-0.785	-0.795	-0.615	-0.791
	p	0.004^***^	0.011^**^	0.102	0.071
CO (Day 0) [mg/m³]	r	-0.284	-0.786	-0.708	-0.823
	p	0.104	0.016^**^	0.011^**^	0.232
PM_10_ (Day 0) [μg/m³]	r	-0.783	-0.698	-0.520	-0.755
	p	0.012^**^	0.029^**^	0.195	0.014^**^

For NO₂, statistically significant negative correlations were found with FVC%pred (r = -0.745, p = 0.019) and FEV₁%pred (r = -0.773, p = 0.017). The SO₂ case showed statistically significant negative correlations with FVC%pred (r = -0.785, p = 0.004) and FEV₁%pred (r = -0.795, p = 0.011). Regarding CO, statistically significant negative correlations were found with FEV₁%pred (r = -0.786, p = 0.016) and FEF_25-75%_​​​​​​​ (%pred) (r = -0.708, p = 0.011). After data processing, statistically significant negative correlations were found between PEF%pred (r = -0.755, p = 0.014) and both FVC%pred (r = -0.783, p = 0.012) and FEV₁%pred (r = -0.698, p = 0.029) for the atmospheric particles PM₁₀.

## Discussion

This study aimed to assess the correlation between air pollution levels and respiratory function parameters in patients with chronic obstructive lung diseases residing in various regions of the Attica Basin, Greece. In the analysis, we used air pollution measurements provided by the Greek Ministry of Environment and Energy's Department of Atmospheric Quality, specifically daily bulletins and monitoring data. Patients with bronchial asthma, COPD, and bronchiectasis were evaluated at the Respiratory Function Laboratory of the First Department of Respiratory Medicine at the Medical School of the National and Kapodistrian University of Athens, for diagnosis and monitoring purposes. The study identified statistically significant negative correlations on examination Day 0 between O₃ concentrations, NO₂ concentrations, SO₂ concentrations, and CO concentrations and respiratory function parameters in both men and women, as well as in smokers and non-smokers. Additionally, statistically significant negative correlations were observed between particulate matter (PM₁₀) concentrations and the parameters peak expiratory flow (PEF%pred) and forced vital capacity (FVC%pred) in the entire sample, including men, women, and smokers, on the same day. Among non-smokers, statistically significant negative correlations were also noted with forced vital capacity (FVC%pred), peak expiratory flow (PEF%pred), and forced expiratory volume in one second (FEV₁%pred) on the same day.

The effects of air pollution, especially ozone, merit high interest, globally. It is well documented that even short-term exposure to increased levels of air pollution has the potential to worsen respiratory symptoms in both healthy individuals and patients with chronic lung conditions such as asthma and COPD. Several studies have shown a potential role for pollutants in the pathogenesis of asthma and COPD [24]. Specifically, elevated O_3_ concentrations are expected to result in more frequent and severe ozone episodes [25], posing an important risk factor for asthma and COPD [26]. Our data are in line with other studies showing a causative correlation between increased ozone levels, and increased school absences, increased visits to emergency rooms, and increased hospital admissions [27-30]. Long-term exposures to ozone have been associated with lower lung function and deteriorated or abnormal lung development in children [31,32]. Recent data suggest a potential causal relationship between long-term exposure to high-level ambient O_3_ and increased risks of adult-onset asthma [33].

Experts predict that climate change will significantly affect ozone (O₃) levels, leading to higher concentrations and altered weather conditions [25]. Air pollution has significant impacts on human health, triggering respiratory symptoms even in healthy individuals and contributing to the development of asthma in both adults and children. The greatest effects are seen in the elderly, children, athletes, and outdoor workers. Women are more affected than men by some pollutants, while men are more affected than women by others [34]. Vulnerable groups, such as those with obesity, diabetes, COPD, asthma, pneumonia, cystic fibrosis, and cardiorespiratory diseases, are particularly affected [34].

Air pollutants, such as nitrogen oxides (NOₓ), sulfur oxides (SOₓ), and ozone (O₃), negatively impact people's quality of life, leading to wage losses, increased morbidity, and elevated mortality. For example, from 2005 to 2007, hospital costs in California were significantly affected by air pollution. Of the total estimated cost of $193 million, $58 million was attributed to exposure to tropospheric ozone (O₃) [35].

These costs place a significant burden on the sustainability of national health insurance systems globally, as well as on the patients themselves. Experts estimate that healthcare costs due to increased ozone concentrations could reach $580 million over the next 30 years, with mortality surpassing two million individuals [36].

The economic and social consequences of air pollution must be acknowledged, as high ozone concentrations led to €220 million in commercial losses in 2005 [37]. Air pollutants such as O₃, CO, and NO₂ have been associated with adverse effects on both morbidity and mortality [38]. Findings from epidemiological studies have prompted revisions to air quality standards and guidelines, with further updates scheduled for the future [39].

The main strength of this study is that it provides real-world data and a large dataset across multiple geographic stations in the Attica Basin, a geographic area that includes almost half of the population of Greece. Another strength is the stratified analysis of the data (gender, smoking status), which gives depth to the findings.

A limitation of the study could be the fact that air pollution measurements were obtained from monitoring stations operated by the Department of Atmospheric Quality of the Greek Ministry of Environment and Energy, all located within the Attica Basin. Therefore, these data primarily reflect this specific geographic area and cannot be generalized. Despite that patients were stable and long-living in the same area of the city, air pollution measurements of the prior three to four days or a week before spirometry might better reflect the causal effect of exposure to lung function testing. Another limitation of the study was that univariate correlation analyses were not corrected for multiple comparisons, thus raising the possibility of a type I error. The lack of a multivariate regression analysis limits the generalizability of our findings. Furthermore, this was an observational cross-sectional study, which limits causal inference, and exposure timing was based on same-day pollution levels, so long-term exposure could not be estimated.

## Conclusions

Air pollution measurements were found to be negatively and statistically significantly correlated with the lung function parameters. Specifically, increased concentrations of air pollutants O₃, CO, SO₂, NO₂, and PM_10 _were significantly associated with lower values of the lung function parameters. These findings highlight the detrimental impact of air pollutants on respiratory health. These data offer a comprehensive understanding of human exposure to air pollution and its impact on health, specifically focusing on the correlation between chronic obstructive lung diseases and the levels of air pollution in the Attica Basin, Greece.
